# Mining the Resource of Cross-Presentation

**DOI:** 10.3389/fimmu.2014.00062

**Published:** 2014-02-27

**Authors:** Anne Hosmalin

**Affiliations:** ^1^INSERM U1016, Institut Cochin, Paris, France; ^2^CNRS UMR8104, Paris, France; ^3^University of Paris Descartes, Paris, France; ^4^Assistance Publique-Hôpitaux de Paris, Hôpital Cochin, Paris, France

**Keywords:** cross-presentation, dendritic cells, immunotherapy, tumors, HIV

(1) In the face of MHC-class I-restricted direct presentation from a live, replicating viral infection – after the groundbreaking discovery of the MHC restriction of T cell responses by Rolph Zinkernagel and Peter Doherty (1) – cross-presentation is indeed a weaker phenomenon (2, 3). Antigen presenting cells can cross-present exogenous antigens from viruses which cannot infect them, allowing anti-viral MHC-class I-restricted, CD8^+^, cytotoxic T cell priming (4). Nevertheless, when direct presentation is available, cross-presentation is dispensable for eliciting a maximal anti-vaccinia virus CD8^+^ T cell response (5).
(2) Demonstration of cross-priming often uses sensitive detection methods requiring the infusion of high numbers of T cell receptor-transgenic mouse T cells (3). However, in many studies, natural CD8 T cell responses to epitopic peptides were induced in mice by cross-priming (6–11).
(3) Dendritic cells (DC) and/or macrophages are indeed key transporters of antigens to secondary lymphoid organs (12–16).
(4) Via class I, DC present phagocytosed antigens from intracellular viruses, bacteria, and other microorganisms, but also from non-replicating microorganisms [HIV inactivated by antiprotease, replication level proven unable to induce direct presentation (17)], apoptotic cells or tumor cell-derived fragments (9), or even live cells [DC purified after culture with live tumor cells, then injected, and the wash-out from these cells, containing potentially contaminating antigen from these tumor cells, is not able to present directly (18)]. This can lead to protective vaccination against tumors (18–20).
(5) Location in secondary lymphoid organs or tertiary lymphatic tissues may indeed be the key to CD8 T cell priming. Any cell type may be able to prime CD8 T lymphocytes when located in lymphatic tissues and correctly activated (21–23). This in turn requires appropriate draining of these cells into lymphatic tissues to provide antigen amounts high enough for cross-presentation, in the presence of the appropriate costimulation and cytokines to induce either immune responses or active tolerance. This conjunction of circumstances may be obtained less rarely with DC than with other cell types, thanks to their high expression of class I molecules, costimulation molecules and cytokines, and their high propensity to transport antigens to lymphatic tissues. This can yield the direct presentation of endogenous epitopes. Why would not it also yield the cross-presentation of exogenous antigens?
(6) During HIV infection, like in LCMV infection, chronic type I IFN production and immune hyperactivation induce immune suppression. Live replicating recombinant vaccine vectors that induce efficient direct presentation are not acceptable for immune therapy in populations with potential immune deficiency, for safety reasons. In addition, these vectors require the use of sequences, which will not mutate like the actual patient’s viral sequences. Why not try and exploit this opportunity to stir the balance of HIV-specific immune responses toward immunity instead of tolerance?
(7) Tumors also favor suppressive mechanisms (negative costimulation molecules like CTLA-4 and PD-1, suppressive cytokines like IL-10, myeloid-derived suppressor cells). After tumor ablation, it may be hoped in the future to restore immune surveillance by anti-suppressive agents and therapeutic vaccination. Recombinant vaccines expressing tumor antigens for direct presentation require the use of sequences, which will not mutate like the actual patient’s tumor antigen sequences. Antigens from dead tumor cells can be crosspresented, even though with a low efficiency, by DC, yielding protection against tumors *in vivo* (in experimental settings that are still artificial but protective) (9); this can be obtained more efficiently using live tumor cells (18). Why not try and exploit this opportunity to stir the balance of antitumoral immune responses toward immunity instead of tolerance?
(8) When major direct antigen presentation is not as blatant as during replicative viral infections, or is not exploitable for safety reasons, the alternative cross-presentation pathway may be exploitable for therapy.
